# Markers to Rapidly Distinguish *Bacillus paralicheniformis* From the Very Close Relative, *Bacillus licheniformis*

**DOI:** 10.3389/fmicb.2020.596828

**Published:** 2021-01-11

**Authors:** Atinuke M. Olajide, Shu Chen, Gisèle LaPointe

**Affiliations:** ^1^CRIFS, Department of Food Science, University of Guelph, Ontario, ON, Canada; ^2^Agriculture and Food Laboratory, Laboratory Services Division, University of Guelph, Ontario, ON, Canada

**Keywords:** Bacillus paralicheniformis, markers, fengycin, dairy, draft genome

## Abstract

As close relatives, *Bacillus paralicheniformis* is often wrongly identified as *Bacillus licheniformis*. In this study, two genetic markers are presented based on *fenC* and *fenD* from the fengycin operon of *B. paralicheniformis* to rapidly distinguish it from *B. licheniformis*. The fengycin operon is one of the few present in *B. paralicheniformis* but absent in *B. lichenformis* up to date. Using these markers, two presumptive *B. paralicheniformis* isolates each were recovered from a set of isolates previously identified as *B. licheniformis* by Matrix-assisted laser desorption ionization–time of flight (MALDI-TOF) or identified only to genus level as *Bacillus* by 16S ribosomal RNA (rRNA) gene sequencing, respectively. Whole genome sequencing of the four isolates confirmed their identity as *B. paralicheniformis* having the closest similarity with *B. paralicheniformis* ATCC 9945a (GenBank: CP005965.1) with a 7,682 k-mer score and 97.22% Average Nucleotide Identity (ANI). ANI of 100% suggests that the four isolates are highly similar. Further analysis will be necessary to determine if finer differences exist among these isolates at the level of single nucleotide polymorphisms.

## Introduction

In the *Bacillus* genus, many species within the assigned groups are very closely related so that 16S ribosomal RNA (rRNA) gene sequences cannot be used to distinguish them (Branquinho et al., 2014a). For instance, in the *Bacillus cereus* group, more than 97% sequence similarity exists among the 16S rRNA genes of *Bacillus anthracis*, *B. cereus*, *Bacillus weihenstephanensis*, *Bacillus thuringiensis*, *Bacillus mycoides*, *Bacillus pseudomycoides*, *Bacillus cytotoxicus*, *Bacillus gaemokensis*, and *Bacillus manliponensis* (Guinebretière et al., 2013). Furthermore, in the *Bacillus pumilus* group, *B. pumilus*, *Bacillus safensis*, *Bacillus altitudinis*, *Bacillus stratosphericus*, *Bacillus aerophilus*, *Bacillus xiamenensis*, and *Bacillus invictae* have over 99.5% similarity in their 16S rRNA gene sequences (Satomi et al., 2006; Liu et al., 2013; Lai et al., 2014; Branquinho et al., 2014b). Likewise, based on the comparative analysis of the 16S rRNA gene, *Bacillus paralicheniformis* strain KACC 18426 (KJ-16^T^) is 99.5% similar to *Bacillus sonorensis* KCTC-13918^T^ and 99.4% similar to *Bacillus licheniformis* DSM 13^T^ (Dunlap et al., 2015).

Due to the close relatedness of *B. paralicheniformis* to *B. licheniformis*, the former has been wrongly identified as *B. licheniformis* up until 2015. The phylogenetic analysis of nine *B. paralicheniformis* and 46 *B. licheniformis* showed that most of the former belonged to lineage P while the latter clustered together in lineage L (Du et al., 2019). However, the researchers observed that a few *B. lichenformis* clustered with *B. paralicheniformis* in lineage P rather than in lineage L. The genome comparative study by Du et al. (2019) revealed differences existing in the operons implicated in secondary metabolite synthesis among many *Bacillus* species. Some examples are the fengycin, paralichenicidin, and bacitracin operons, encoded by the genomes of *B. paralicheniformis* but absent in those of *B. licheniformis* analyzed to date (Harwood et al., 2018; Du et al., 2019).

**Figure 1:**
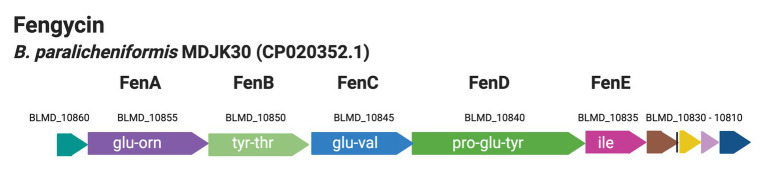
Genetic organization of the cluster coding for fengycin biosynthesis from the genome of *B. paralicheniformis* MDJK30 (Du et al., 2019).

Fengycins belong to a group of non-ribosomally synthesized antifungal lipopeptides, produced by certain strains of *Bacillus* species, such as *B. subtilis*, *B. velezensis*, *B. paralicheniformis*, and *B. amyloliquefaciens* (Steller et al., 1999; Stein, 2005; Harwood et al., 2018; Du et al., 2019). Also, fengycin lowered the numbers of *Staphylococcus aureus* in the human intestine (Piewngam et al., 2018). Fengycin synthetase consists of five non-ribosomal peptide synthetases (NRPSs) FenA – FenE encoded by *fenA* – *fenE* (Figure 1; Vanittanakom et al., 1986; Steller et al., 1999; Chen et al., 2007). Fengycin is a cyclic lipodecapeptide (CLP) containing a β-hydroxy fatty acid side chain of up to 19 carbon atoms (Steller et al., 1999) while the peptide portion contains at least four amino acids (Steller et al., 1999). The structure and function of the fengycin synthetase of *B. subtilis* origin have been reviewed previously by Steller et al. (1999).

Although Dunlap et al. (2015) attempted to differentiate *B. paralicheniformis* from *B. licheniformis* based on 16S rRNA gene phylogenetic and phenotypic analyses; there are no genetic markers that have been used to rapidly achieve this. To adequately identify *Bacillus* species, known to cause spoilage in foods including dairy, it is important to develop markers that work rapidly and efficiently in distinguishing these close relatives. Hence, *B. paralicheniformis* specific markers were designed based on the *fenC* gene (7,640 bp) and the *fenD* (10,780 bp). The markers were used to screen a collection of isolates previously identified as *B. licheniformis* or *Bacillus* spp. by 16S rRNA gene sequencing, matrix-assisted laser desorption ionization–time of flight (MALDI-TOF), or *B. licheniformis* specific primers. To the best of our knowledge, dairy *B. paralicheniformis* isolates of Canadian origin have not yet been reported.

## Materials and Methods

### Isolate Identification

Three hundred and nineteen (319) isolates were obtained after spore pasteurization (80°C for 12 min) or laboratory pasteurization (63°C for 30 min) of samples (raw milk, HT-milk, whey, curds, and environmental swabs) from a Cheddar cheese making plant and incubation on BHI agar (Oxoid) at 30°C for 48 h (aerobic). The identification was carried out by Sanger sequencing of the 16S rRNA gene, MALDI-TOF mass spectroscopy or *B. licheniformis* specific primers by de Almeida (2014). Sanger sequencing of at least 750 bp of the 16S rRNA gene was carried out using the FD1 forward primer (5' AGAGTTTGATCCTGGCTCAG-3') and the RD1 reverse primer (5' AAGGAGGTGATCCAGCCGCA-3'; Weisburg et al., 1991). Colony PCR was carried out with the condition as follows: 94°C for 2 min, 35 cycles of 94°C for 15 s, 55°C for 30 s, 72°C for 1.5 min and finally, and 72°C for 10 min. After amplification, the amplicons were separated by agarose gel electrophoresis (1% w/v, 70 v for 30 min) and visualized using a ChemiDoc Imaging System (Bio-Rad, Canada). Once the appropriate bands were purified, the products were sequenced at Eurofins (Canada). The sequences obtained per isolate were assembled (using DNA Baser v4) and compared against the National Center for Biotechnology Information (NCBI) database using the Ribosomal Database Project (Michigan State University) to obtain identification (> 99% similarity to species level).

Matrix-assisted laser desorption ionization–time of flight was used to identify isolates at the Animal Health Laboratory, University of Guelph (Guelph, ON, Canada) on the MALDI Biotyper (Bruker, Canada) with the software Compass v 4.1.80 (PYTH) 1022017-08-226_04-55-52. Each isolate to be identified was struck on a plate (Brain Heart Infusion agar at 30°C for 1 day) to obtain single and distinct colonies. After incubation, the isolates were maintained at room temperature until they were transported to the MALDI-TOF analysis facility. A bacterial colony was directly transferred to a 96-spot stainless steel target plate. One microliter of HCCA matrix (*α*-Cyano-4-hydroxycinnamic acid) was applied to each spot and left to dry (at room temperature). One microliter 70% formic acid was applied to the spot and allowed to dry followed by the addition of another 1 μl HCCA matrix after which it was left to dry. Identification was completed using a Bruker MALDI Biotyper and the software “Compass version 4.1.80 (PYTH) 1022017-08-226_04-55-52.” Calibration of each run was completed by applying a Bacterial Test Standard (BTS) to each target. The BTS was an *Escherichia coli* extract spiked with two high molecular weight proteins that was developed for the quality control process of the MALDI Biotyper System. The specific composition of the BTS covered the entire mass range of proteins used for precise identification of microorganisms. This control was run at every MALDI identification procedure, and the calibration must pass in flex control with a score greater than 2.0. A MALDI-TOF spectrum was automatically generated by the software and instantly matched against the reference library to give identification. For taxonomy allocation, if an organism obtained a score (*x*): *x* < 1.7, the ID was reported as “not reliable identification”; a score between 1.7 ≤ *x* ≤ 1.99 was reported to genus level only and with a score *x* ≥ 2.0, the ID was reliably reported to species level.

Lastly, four *B. licheniformis* specific primer sets (in pairs per PCR) were applied to crude DNA from *Bacillus* isolates with undetermined species as described by de Almeida (2014) (Table 1) using DNA from *B. licheniformis* ATCC 14580 and *B. paralicheniformis* KACC 18426 as positive and negative control, respectively. The PCR reaction mixture (23.5 μl) contained 1x PCR Supermix (Invitrogen), 0.25 μl (12.5 μM) of each primer, and 10 μl of template DNA. The amplification was as follows: initial denaturation for 5 min at 95°C, 35 cycles of 30 s at 95°C, 30 s at 58°C, and 90 s at 72°C followed by a final extension step for 10 min at 72°C. The amplification products were separated on a 2% agarose gel at 100 V for 35 min. Isolates for which amplicons were obtained by all four primer sets were assigned to *B. licheniformis*.

**Table 1:** *Bacillus licheniformis* specific primer sequences and amplification conditions.

PCR	ORF	Products	Marker	Primers	Primer sequence (5' – 3')	Amplicon size (bp)	Limitation (observed during the *in-silico* primer verification *via* NCBI)
PCR 1	BL00303	hypothetical protein	BL5B	Forward	CGCTCACCATATGCACAGCTCT	332	Amplified at least 1 *B. paralicheniformis* genome
				Reverse	CGGTTTATCGCTTGAGACYCGG		
	serA2	3-phosphoglycerate dehydrogenase	BL8A	Forward	TCACAACCCGTTGACGACAA	247	Amplified at least 1 *B. paralicheniformis* or *B. glycinifermentans* genome
				Reverse	CGTGTCCGAGTGTGCGTTATAT		
PCR 2	BLi00806	hypothetical protein	BL13C	Forward	TTGTGCGTATCTCCGGGCCA	376	Amplified at least 1 *B. paralicheniformis* genome
				Reverse	AGGCATTGTCCCGATGGTGG		
	ligD	ATP-dependent DNA ligase	BL18A	Forward	GTCAACGACACAATTTCCCCGT	216	Amplified about 6 *B. paralicheniformis* genomes with 2 mismatched nucleotides
				Reverse	AGCTCCCTCAGGCGGCAATT		

Letter Y = nucleotides T/C. Marker BL5B was not present in all genomes deposited as *B. licheniformis* in National Center for Biotechnology Information (NCBI) while marker BL8A gave unspecific amplification with other *Bacillus* species.

### Identification of the Fengycin Operon in the *B. paralicheniformis* Genome

The genes making up the fengycin operon were searched using BLAST within the six *B. paralicheniformis* whole genomes present in the NCBI GenBank (NZ_CP023666.1, NZ_CP023168.1, NC_021362.1, NZ_CP020352.1, NZ_CP023665.1, and NZ_CP033389.1). Genes were located using their arrangement in the genome of strain MDJK30 as a reference. The two longest genes (*fenC* and *fenD*) of the operon were chosen for designing primers. The sequences of these two genes from the six genomes listed above were aligned (MEGA 7; default settings) and observed to be 100% identical.

### Primer Design and PCR

A primer set for each of *fenC* and *fenD* genes were designed (using PrimerQuest tool on IDT.com) based on the *B. paralicheniformis* MDJK30 genome sequence (GenBank: NZ_CP020352.1). Once specificity for *B. paralicheniformis* was ascertained *in silico*, primers were synthesized by Eurofins (Canada) and the PCR conditions were optimized using *B. paralicheniformis* KACC 18426.

Crude DNA was extracted from all the isolates identified as *B. licheniformis* isolates and *Bacillus* spp. isolates using Instagene matrix according to the manufacturers’ recommendations (Bio-Rad, Canada) and screened (Table 2). The PCR reaction mix of 23.5 μl contained 1× PCR Supermix (Invitrogen), 10 μM (0.5 μl) each of forward and reverse primers and 10 μl of template DNA (approx. 10–100 ng). The PCR cycle was carried out on Biometra professional thermocycler as follows: 2 min at 95°C, 40 cycles of 30 s at 95°C, 30 s at 53°C, and 1.5 min at 72°C, followed by a final extension step at 72°C for 5 min.

**Table 2:** PCR primer sequences and amplification conditions for *fenC* and *fenD.*

Gene	Primer name	Direction	5' to 3'	Start	Stop	Length	Tm	GC %	Amplicon length
*fenC*	FenCf	Forward	CCGCAAGACTGAGAGAAATA	6,204	6,224	20	59	45	446
	FenCf	Reverse	CGACGACCAAATGATGAATG	6,630	6,650	20	59	45	
*fenD*	FenDf	Forward	GGATAGTCCTGGTGTTCATAG	6,695	6,716	21	59	47.6	803
	FenDr	Reverse	CAGAGAGTGGAAGCTGTATT	7,478	7,498	20	59	45	

### Confirmation of Presumptive *Bacillus paralicheniformis* Isolates

The isolates which gave amplicons (of correct sizes) with the two sets of *B. paralicheniformis* specific primers were sequenced using the Sanger method which was carried out at Eurofins (Canada). Also, whole genome sequencing of the presumptive *B. paralicheniformis* isolates was carried out for confirmation of their identity and diversity. For each isolate, genomic DNA was extracted from a single colony (picked from freshly prepared plates) using the Ultraclean Microbial DNA Isolation Kit (Qiagen, Canada), following the manufacturer’s protocol. The sequencing was carried out at the Laboratory Services, University of Guelph (Guelph, Ontario). The sequence library preparation was done using the Nextera DNA Flex library preparation kit (Illumina, Canada) following the manufacturer’s protocol. A MiSeq sequencer was used for sequencing with a MiSeq V2 reagent kit (Illumina) and 2 × 250 paired-end cycles, according to the manufacturer’s recommendation.

The MiSeq sequencer system software v3.1 (Illumina) was used to process the raw sequence reads. Furthermore, short sequences were filtered using FastQC 1.0.0 in BaseSpace.^1^ The sequences that passed a quality score of 30 were assembled *via de novo* assembly following an overlap-layout-consensus method using SPAdes v3.9.0 Genome Assembler (Bankevich et al., 2012) to generate contigs and scaffolds. Bacterial Analysis Pipeline v1.0.4 was used to predict the bacterial species following a k-mer based approach (Larsen et al., 2014). An Average Nucleotide Identity (ANI) calculator on EZBioCloud (https://www.ezbiocloud.net/; Yoon et al., 2017) was used to determine the percentage of nucleotide identity. NCBI Prokaryotic Genome Annotation Pipeline (PGAP) v4.10 was used to predict, name, and annotate genes using the best-placed reference protein set method (Tatusova et al., 2016). Default parameters were used for all software.

### Maximum Likelihood Dendrogram

Using the genomes of *B. subtilis* (168 and ATCC 13952), *B. velezensis* (UCMB5033 and BZB42), *B. amyloliquefaciens* (IT-45 and DSM 7), and *B. paralicheniformis* MDJK30 and a presumptive *B. paralicheniformis* isolate, a maximum likelihood dendrogram was constructed. Consensus sequences of the fengycin operon were extracted from each genome and aligned in the CLC workbench genomics (v10.0), after which the tree was created using MEGA (K2 + I model).

## Results

### Identification of *Bacillus* Isolates

**Figure 2:**
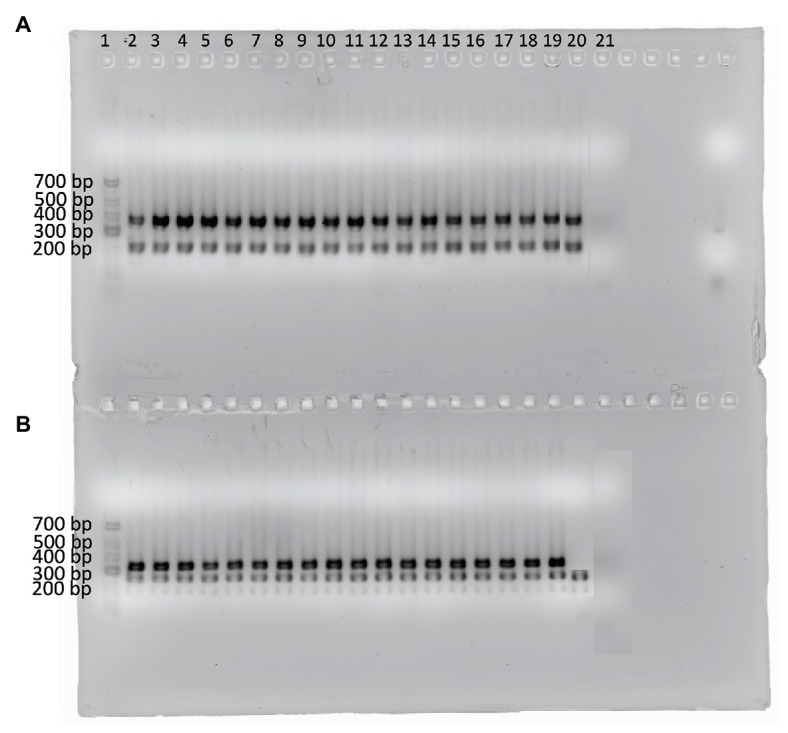
Agarose gel showing the amplicons from 17 isolates identified as *B. licheniformis* using the four *B. licheniformis* specific markers (de Almeida, 2014). **(A)** Multiplex 1: primer sets 18A and 13C; **(B)** Multiplex 2: primer sets 5B and 8A. Lane 1 contains the GeneRuler Low range DNA ladder (ThermoScientific). Lanes 2–18 represent the additional *B. licheniformis* isolates while lane 19 contains *B. licheniformis* ATCC 14580 (positive control), lane 20 contains *B. paralicheniformis* KACC 18426 (negative control), and lane 21 contains the no template control.

Out of 319 isolates, 124 were identified as *B. licheniformis* while 116 were identified to the genus level as *Bacillus* by 16S rRNA sequencing and MALDI-TOF. Using the species-specific primers, 17 additional *B. licheniformis* isolates were obtained (Figure 2) out of the 116 isolates previously identified to the genus level as *Bacillus*. However, the negative control, *B. paralicheniformis* KACC 18426, was amplified by three out of the four primer sets (BL13C, BL18A, and BL8A). Overall, 141 *B. licheniformis* and 85 *Bacillus* spp. were identified, resulting in a total of 226 isolates for screening.

### Detection of *B. paralicheniformis* Isolates

**Figure 3:**
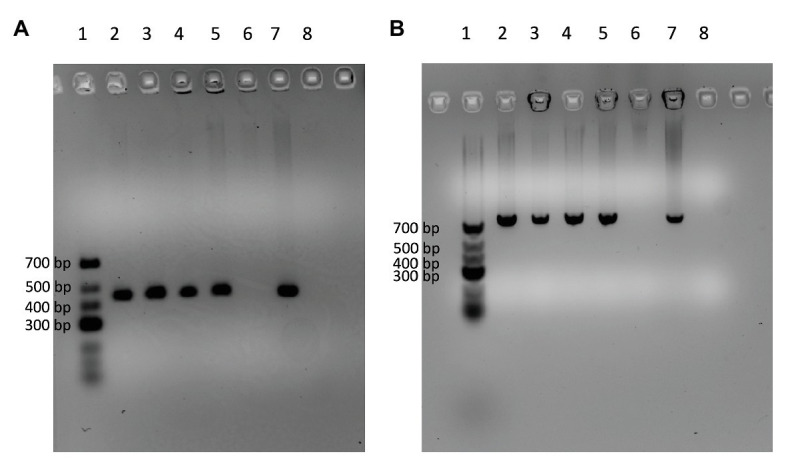
Agarose gels showing four isolates (Lanes 2–5) which were amplified by the DNA markers [*fenC* **(A)** and *fenD* **(B)**] designed specifically for *B. paralicheniformis* using *B. licheniformis* ATCC 14580 as negative control (Lane 6) and *B. paralicheniformis* KACC 18426 (NRRL – B65293) as positive control (Lane 7). Lane 8 contains the no-template control of the PCR reaction. Lane 1 contains the GeneRuler Low range DNA ladder (ThermoScientific).

Out of the 226 isolates tested, four (9MF010A, 9MF10B, 7CS50, and 7WI3) gave amplicons with both the *fenC* and *fenD* gene primer sets (Table 3). On all occasions, DNA from *B. licheniformis* ATCC 14580 (negative control) and the no-template control were not amplified by both sets of primers (Figure 3). Sanger sequencing of the amplicons from *fenD* confirmed that they were 99% identical to *B. paralicheniformis* ATCC 9945a as top match.

**Table 3:** Isolates newly identified as *Bacillus paralicheniformis*.

Isolate code	Isolation source	Isolation method	Initial method of identification	Initial identity
7WI3	Whey	ASC	16S rRNA gene Sanger sequencing	*Bacillus* spp.
9MF10A	HT-milk	ASC	MALDI-TOF	*B. licheniformis*
9MF10B	HT-milk	ASC	MALDI-TOF	*B. licheniformis*
7CS50	Cheese curd	ASC	16S rRNA gene Sanger sequencing	*Bacillus* spp.

HT-milk refers to heat-treated (thermized) milk.

### Whole Genome Sequencing of Presumptive *Bacillus paralicheniformis* Isolates

**Figure 4:**
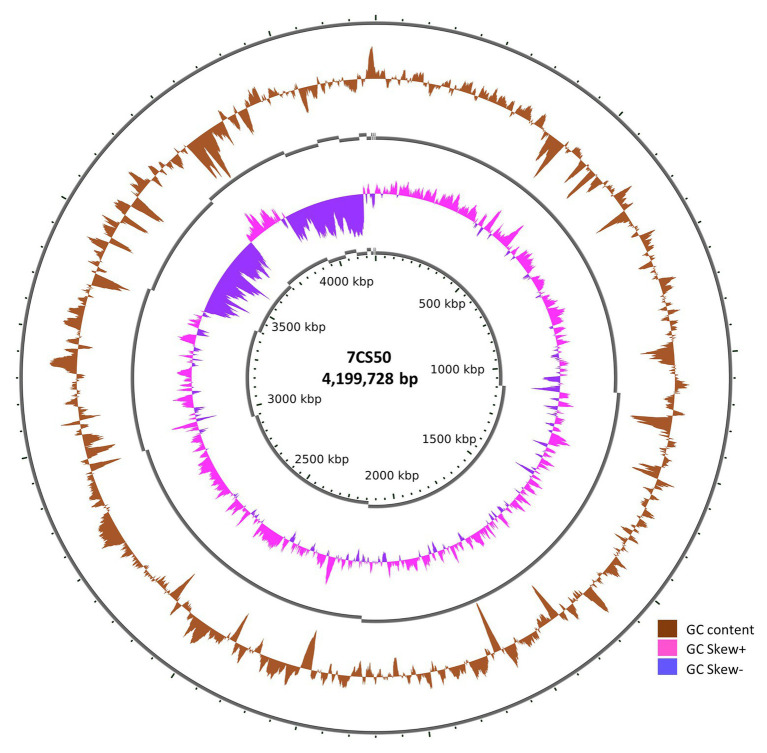
The circular genome of *B. paralicheniformis* 7CS50 using the CGViewer and a command line (https://github.com/paulstothard/cgview) for multiple contigs (Grant and Stothard, 2008).

**Figure 5:**
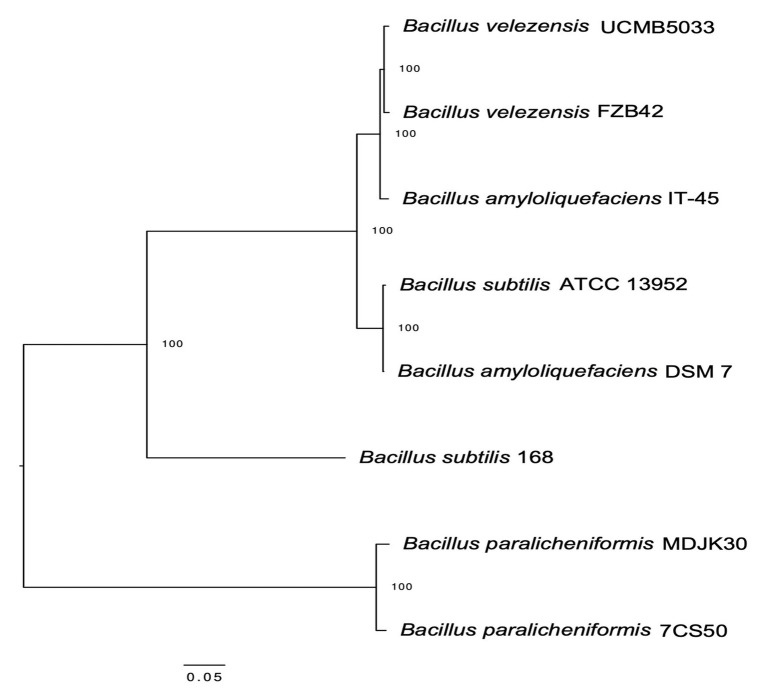
Maximum likelihood dendrogram based on the fengycin operon alignment from *B. paralicheniformis* 7CS50 and seven other *Bacillus* reference sequences *via* MEGA (K2 + I model) using the CLC workbench genomics v 10.

Whole genome sequencing of the four *B. paralicheniformis* isolates showed an ANI of 100%, therefore, only one genome (7CS50) is presented here. The genome was closest to *B. paralicheniformis* ATCC 9945a (GenBank: CP005965.1) with a k-mer score of 7,682 and ANI of 97.22% was identified as *B. paralicheniformis*. With 137x coverage, 2,302,435 reads were generated for strain 7CS50 which were trimmed to 250 bp and assembled into 26 contigs. Strain 7CS50 has an estimated genome size of 4,199,730 bp and a 46.07% GC content (Figure 4). In total, 3,981 protein-coding sequences, 111 RNAs and 4,217 genes were found. The fengycin operon in *B. paralicheniformis* 7CS50 is closest in overall nucleotide identity to strain MDJK30 (97%) and farthest from the other species (78–87%; Figure 5).

## Discussion

Fengycin was selected for the design of the *B. paralicheniformis* specific primers due to the ease of locating the operon in at least five publicly available genomes coupled with the ease of primer design. As most relevant literature target *B. licheniformis* for marker design, developing markers based on *B. paralicheniformis* should be explored. Of the five large proteins in the fengycin operon, only *fenC* and *fenD* were present in the six genomes with complete genome sequence data in the NCBI GenBank database.

The four isolates which were amplified by the designed primers were confirmed to be *B. paralicheniformis* using *B. paralicheniformis* ATCC 9945a as the reference genome. The four genomes encode the fengycin A–D operon, specific to *B. paralicheniformis* (Du et al., 2019). Two of the isolates were obtained from winter cheese and whey samples from separate vats while the other two were from a heat-treated (HT) milk samples collected in spring, all from a cheese making plant. These isolates were obtained after spore pasteurization (80°C for 12 min) of dairy samples obtained from a cheese processing system during the aerobic spore former count (ASC). These four isolates were highly similar as their sequences showed an ANI score of 100%. *Bacillus licheniformis* ATCC 14580 DNA was not amplified by the primer sets, implying that the sets were indeed specific for *B. paralicheniformis*.

*Bacillus paralicheniformis* specific markers have not been reported to date, to the best of our knowledge. These markers provide a fast detection of *B. paralicheniformis* among presumptive *B. licheniformis* isolates or even isolates identified to genus level as *Bacillus*. These markers are very promising as they provide a better approach to distinguishing this closely related *Bacillus* in comparison to previous phylogenetic and the phenotypic approaches. The four new *B. paralicheniformis* isolates had been previously identified as *B. licheniformis* by MALDI-TOF or as *Bacillus* spp. by Sanger sequencing of the 16S rRNA gene. It can be inferred that these approaches cannot adequately identify this bacterial species, as seen with many other close relatives among the genera *Bacillus*, *Anoxybacillus*, and *Geobacillus* (Randazzo et al., 2009; Weng et al., 2009; Burgess et al., 2010; Branquinho et al., 2014a). The misidentification of *B. paralicheniformis* by MALDI-TOF may have been due to either the quality and size of the protein database itself or due to the instability of the proteins produced by the isolates (Stîngu et al., 2008; Seng et al., 2009; Starostin et al., 2015). Adding the protein profile of these isolates to the MALDI-TOF database will improve future discrimination of the species using this method. Although 16S rRNA gene sequencing has been the gold standard for bacterial identification and for inferring phylogenetic relationships, it shows insufficient variation between closely related species (Zeigler, 2005). Similarly, species-specific primers of *B. licheniformis* that are not designed based on operons lacking in *B. paralicheniformis* will likely amplify the latter. This probably means that many of the primers previously designed specifically for *B. lichenformis* might also detect *B. paralicheniformis*. Even though *fenC* and *fenD* are not housekeeping genes which are alternative options for providing accurate taxonomic resolution of close relatives, they may be suitable to provide presumptive species identification. These markers could be used singly or in duplex to distinguish these two closely related species.

Across *Bacillus* species that possess a fengycin operon, the level of homology in terms of protein identity was low, ranging from 62 to 74%, indicating divergence from a common ancestor rather than horizontal transfer of these genes (Du et al., 2019). The fengycin operon from strain 7CS50 shows less than 90% nucleotide identity with the fengycin operons from seven other *Bacillus* species, which also suggests divergence, not horizontal transfer. Horizontal gene transfer (HGT) can occur in three main ways: transformation, transduction, and conjugation (Thomas and Nielsen, 2005). Genes (even if they are not accompanied by mobile elements) can be integrated into genomes if there is an active recombination system in the strain. Similarly, cells must be competent to take up DNA and integrate it into their genome (Johnston et al., 2014). Therefore, if *fenC* or *fenD* were transferred to *B. licheniformis*, then the strain would become indistinguishable from *B. paralicheniformis* with these markers. To mitigate this problem, it would be prudent to combine these markers with additional types of taxonomic identification or other markers based on secondary metabolites such as paralichenicidin (specific to *B. paralicheniformis*) or lichenicidin (specific to *B. licheniformis*). There appears to be a lower risk of HGT with the diverse polyketide synthase (pks) and NRPS-type operons (Grubbs et al., 2017), even though they are often found on genomic islands. Other approaches can be considered, such as Single Nucleotide Polymorphisms (SNPs) or a k-mer based approach as well as the use of smaller individual genes.

Structures, such as transposons, genomic islands, and integrases could suggest the possibility of HGT (Bose and Grossman, 2010). At this time, an *in silico* search using the IslandViewer online tool (v4 – www.pathogenomics.sfu.ca/islandviewer/query.php) predicted no genomic island close to the fengycin operon in the three *B. paralicheniformis* genomes analyzed (NZ_CP020352.1, NZ_CP023168.1, and NZ_CP023666.1; Bertelli et al., 2017). However, in the *B. amyloliquefaciens* FZB42 genome (now *B. velezensis* FZB42), there is a Tn1546 transposase encoded close to the fengycin operon located on genomic island 10 (Rückert et al., 2011). Similarly, the *bmy* (bacillomycin D) operon (located on a genomic island) is closely inserted by the fengycin operon in the FZB42 genome (Koumoutsi et al., 2004). A low (74%) nucleotide similarity between the fengycin-related gene (*fenD*) in *B.velezensis (amyloliquefaciens)* FZB42 and *B. paralicheniformis* MDJK30 suggests that they are only distantly related. For these markers to remain relevant, the possible occurrence of horizontal gene transfer of specific fengycin operons from *B. paralicheniformis* to *B. licheniformis* accompanied by recombination should continue to be monitored.

## Conclusion

Two markers for the reliable and quick differentiation of *B. licheniformis* from *B. paralicheniformis* were established. They can be useful for presumptive identification of *B. paralicheniformis*. A new dairy *B. paralicheniformis* strain of Canadian origin was discovered using the *fenC* and *fenD* markers. Unlike lanthipeptides, such as bacitracin, the operons encoding fengycin-type NRPS metabolites do not show any evidence of HGT as yet but display a significant level of diversity among *Bacillus* species. The draft genome assembly of strain 7CS50 provides more data for future research on the species such as exploring the genomic diversity and functions of dairy *B. paralicheniformis* strains in comparison with those obtained from other environments.

## Data Availability Statement

The draft genome data were deposited at DDBJ/EMBL/GenBank (BioProject ID PRJNA580480) under the accession number WLVZ00000000 with SRA accession number SRX7224652 (Olajide et al., 2019).

## Author Contributions

AO: methodology, investigation, writing, original draft and revising, formal analysis, and visualization. SC: supervision, resources, formal analysis, writing, reviewing, and editing. GL: conceptualization, supervision, project administration, funding acquisition, resources, writing – review and editing, and data curation. All authors contributed to the article and approved the submitted version.

### Conflict of Interest

The authors declare that the research was conducted in the absence of any commercial or financial relationships that could be construed as a potential conflict of interest.

## Footnotes

^1^https://basespace.illumina.com
